# AI-Assisted Prior Authorization in Traditional Medicare: What the Wasteful and Inappropriate Service Reduction (WISeR) Model Must Demonstrate

**DOI:** 10.1016/j.ajmo.2026.100136

**Published:** 2026-06-19

**Authors:** Henry Bair

**Affiliations:** Wills Eye Hospital, Philadelphia, Pa

**Keywords:** Artificial intelligence, Coverage determinations, Health equity, Medicare, Prior authorization, Utilization management

Prior authorization is often framed as an administrative nuisance, but it is also a policy instrument: it defines how quickly patients can access covered care, how clinicians document medical necessity, and how disagreements are resolved. Used well, prior authorization can curb low-value or unsafe utilization and reduce fraud. Used poorly, it can delay care, increase clinician burden, and create inequities that are hard to see and harder to reverse.

In 2026, prior authorization will become more visible in traditional (fee-for-service) Medicare through the Centers for Medicare & Medicaid Services (CMS) Innovation Center’s Wasteful and Inappropriate Service Reduction (WISeR) Model. WISeR will “harness enhanced technologies,” including artificial intelligence (AI) and machine learning, along with human clinical review, to streamline medical necessity review for a preselected set of items and services vulnerable to fraud, waste, and abuse.^1^ The distinction matters: WISeR is best described as AI-assisted (or AI-supported) prior authorization and prepayment review, because CMS states that recommendations for nonpayment will be determined by appropriately licensed clinicians applying standardized procedures, and that participating companies must have clinician expertise to conduct medical reviews.^1^^,^^2^

Much of the early debate about WISeR has focused on whether traditional Medicare should adopt prior authorization at all. That question is important, and it has already been raised in prominent medical journals.^3^^,^^4^ A more actionable question is what WISeR must *prove* for AI-assisted utilization management to be considered legitimate in Medicare’s baseline program. The answer cannot be limited to savings. It must include whether the program can deliver value (patient-relevant outcomes), procedural fairness (contestable determinations), and equity (no amplification of structural disparities) at scale. This commentary focuses on two underdiscussed WISeR design features, AI-supported workflow decisions and postservice, prepayment review, and offers three implementation-ready additions to strengthen legitimacy and learning value.

The postservice prepayment pathway is particularly consequential: it may not delay the procedure itself, but it can delay reimbursement. If payment holds are prolonged or unpredictable, practices may restrict scheduling, require deposits, or avoid offering certain services—creating access barriers that operate through cash-flow risk rather than explicit coverage denial.

## What WISeR Targets and Why That Choice Is Defensible

WISeR will run from January 1, 2026, through December 31, 2031, in six states (Arizona, New Jersey, Ohio, Oklahoma, Texas, and Washington).^1^^,^^2^ For included services, clinicians may submit a prior authorization request before the service or furnish the service and have the claim routed for postservice, prepayment medical review before payment. WISeR excludes inpatient-only services, emergency services, and those that would pose significant risk if substantially delayed.

CMS has been explicit about why the included services were selected: they have publicly available coverage criteria, are typically elective and not first-line for a condition, may raise patient safety concerns if delivered inappropriately, and are of sufficient volume or dollar value with prior reports of potential fraud, waste, or abuse.^2^ Examples include skin and tissue substitutes, electrical nerve stimulator implants, and knee arthroscopy for knee osteoarthritis.^1^

## What CMS Already Plans to Measure—and What Remains Missing

Some critiques of WISeR understate the guardrails CMS has already specified. CMS states that payment adjustments will be tied to performance measures across three categories, including process quality (nonaffirmations and favorable appeals), experience (timeliness and clarity of explanation), and clinical quality outcomes (use of alternative services and evidence of ongoing urgent need).^1^ In addition, the operational guide emphasizes that “appropriately trained clinicians” with subject matter expertise will be used to verify that requested or furnished services meet Medicare coverage criteria.^2^ These elements are meaningful guardrails.

The remaining risk is whether these metrics are sufficiently patient-centered, transparent for external scrutiny, and capable of attributing harms across CMS, vendors, clinicians, and patients.

## How ‘AI-Assisted’ Prior Authorization Can Still Create New Failure Modes

Even when a clinician makes the final determination, AI-assisted workflows can powerfully shape decisions. In practice, vendors can use algorithms to extract and standardize documentation, triage requests for additional scrutiny, or flag likely noncompliance when key elements appear absent. The model may never be labeled “denial automation,” yet it can become the default gatekeeper through speed, scale, and reviewer dependence on algorithmic outputs.

A key unresolved question is what data such systems learn from. Claims and historical billing data are attractive because they are abundant, standardized, and linked to payment. But billing patterns encode differences in access and documentation: specialist availability, site-of-care resources, language barriers, and prior authorization literacy. If an algorithm uses prior utilization, payment history, or prior denial patterns as proxies for appropriateness, it can reproduce inequities even when the written coverage criteria are neutral. The experience of population-health-risk algorithms is instructive: when cost is used as a proxy for need, bias can be substantial, systematically underidentifying illness burden in historically underserved groups.^5^ AI-assisted utilization management risks analogous problems if “what got paid before” becomes a training signal for “what should be paid now.”

A related risk is that AI-assisted review could normalize sequencing requirements that are administratively simple but not always clinically justified. In prescription drug coverage, “step therapy” or “fail-first” policies require patients to try and fail one or more insurer-preferred therapies before receiving the requested treatment; such policies have prompted state-level reforms requiring exceptions when a prerequisite treatment is ineffective, contraindicated, previously failed, or when the patient is stable on the current therapy.^6^ Although WISeR targets services rather than outpatient drugs, the same principle applies: nonaffirmations should not rest on vendor-specific “try first” logic unless that sequence is grounded in Medicare coverage criteria or evidence-based guidelines and includes patient-specific exceptions.

## The Shared-Savings Design Shifts Responsibility—Clinically and Ethically

WISeR introduces a financing structure that can alter behavior at the margins. CMS states that participating companies will receive a percentage of reductions in spending attributable to reduced wasteful or inappropriate care, which is functionally a shared-savings arrangement.^1^ This aligns incentives with stewardship, but it can also create pressure toward excessive documentation demands or overly aggressive review if oversight is weak.

Equally important, WISeR reallocates responsibility: patients bear delay and disruption, clinicians bear administrative work and reputational risk, vendors control workflow design, and CMS bears policy legitimacy when beneficiaries experience “Medicare” as the decision-maker. Procedural fairness is therefore not merely an ethical preference; it is a prerequisite for trust.

## A More Informative WISeR: Three Additions That Would Raise Legitimacy and Learning Value

Professional societies have raised similar concerns; the American College of Physicians supported reducing waste but warned that outsourcing review functions to AI could introduce opacity, bias, and insufficient accountability.^7^ WISeR will generate better evidence and be more likely to earn clinician and beneficiary trust if CMS adds three concrete elements.

• *Disclose the role of AI in determinations (triage vs recommendation vs automation).*

“AI-assisted” should be defined operationally. CMS should require each WISeR participant to disclose, at least to CMS and, ideally, publicly, whether AI is used for documentation extraction, triage, or for substantive recommendations, and to confirm that nonaffirmations require clinician review.

• *Create an auditable “denial trace” that makes contestability real and distinguishes evidence gaps from sequencing rules.*

A denial that cannot be acted upon is functionally a barrier. Every nonaffirmation should map directly to the relevant Medicare coverage criterion and identify the missing element(s) in a structured way. At minimum, each nonaffirmation should include:

• the specific coverage authority (eg, national or local coverage determination) and criterion invoked;
• the exact criterion element not met;
• the missing data fields or documentation;
• acceptable examples of supporting evidence;
• whether the decision reflects missing documentation, medical necessity, or a required sequence of prior treatments and/or services;
• any patient-specific exceptions, such as contraindication, prior failure, expected ineffectiveness, or stable response to current therapy; and
• clinician reviewer attestation.

This structure converts “clarity of explanation” into an auditable artifact and enables external assessment of whether particular reasons (eg, missing fields or “try first” requirements) disproportionately drive nonaffirmations across populations or practice settings.

• *Expand value measurement beyond spending and utilization to include patient-relevant outcomes.*

WISeR’s “clinical quality outcomes” include use of alternative services and evidence of ongoing urgent need.^1^ Those are reasonable claims-based proxies, but they do not capture the full meaning of value. For some targeted services, the most important outcomes include pain, function, mobility, and quality of life, which are domains that claims data poorly represent.

For WISeR’s postservice, prepayment review pathway, value assessment should also include payment-flow measures: time from service to claim payment, frequency and duration of payment holds, and changes in practice participation (eg, reduced provision of targeted services, increased patient deposits, or shifts in site of care). These are plausible mechanisms by which utilization management can reduce access without increasing formal denial rates.

These outcomes should be stratified to detect disparities and interpreted with appropriate context (eg, recognizing that low appeal rates may reflect reconsideration, substitution, or appeal burden rather than uniformly appropriate denials).^8^^,^^9^

## Looking Beyond WISeR

WISeR is a limited demonstration, but it is also a precedent. If AI-assisted prior authorization is normalized in traditional Medicare, similar approaches are likely to diffuse—through private insurers, state Medicaid programs, and Medicare itself—because administrative technologies scale more easily than the expansion of the clinical workforce. WISeR, therefore, should be treated as a high-stakes test of whether utilization management can be modernized *without* weakening transparency, fairness, and equity. The more explicit CMS is about what algorithms do, the more contestable determinations are for clinicians and beneficiaries, and the more patient-centered outcomes are included in evaluation, the more likely WISeR will generate learning rather than distrust.

## CRediT authorship contribution statement

**Henry Bair:** Writing – original draft.

## Declaration of competing interest

The author does not have any conflicts of interest with the subject matter in the manuscript. The author is solely responsible for the content of this manuscript.
